# Therapeutic effects of extracellular vesicles derived from mesenchymal stem cells primed with disease-conditioned-immune cells in systemic lupus erythematosus

**DOI:** 10.1186/s13075-024-03435-1

**Published:** 2024-11-18

**Authors:** Eun Wha Choi, Il Seob Shin, I-Rang Lim, Jihye Lee, Bongkum Choi, Sungjoo Kim

**Affiliations:** 1 Department of Veterinary Clinical Pathology, College of Veterinary Medicine & Institute of Veterinary Science, Kangwon National University, 1 Kangwondaehak-gil, Chuncheon, Gangwon-do 24341 Republic of Korea; 2 Bioanalysis Center, GenNBio Inc, 700, Daewangpangyo-ro, Bundang-gu, Seongnam-Si, Gyeonggi-do 13488 Republic of Korea; 3 GenNBio Inc, 80, Deurimsandan 2-ro, Cheongbuk-eup, Pyeongtaek-si, Gyeonggi-do 17796 Republic of Korea; 4 Current address: Department of Surgery, Cheju Halla General Hospital, 65, Doryeong-ro, Jeju-Si, Jeju-do 63127 Republic of Korea

**Keywords:** Autoimmune diseases, Conditioned media, Extracellular vesicles, Mesenchymal stem cell, Systemic lupus erythematosus

## Abstract

**Background:**

Systemic lupus erythematosus (SLE) is an incurable chronic autoimmune disease of unknown etiology. Therefore, the development of new treatments is urgently needed. This study aimed to investigate the therapeutic effects of extracellular vesicles (EV) derived from immortalized mesenchymal stem cells (iMSCs) primed with conditioned media obtained from disease-conditioned immune cells (CM-EV) and iMSC-derived EV (ASC-EV) in a murine model of SLE.

**Methods:**

Female NZB/W F1 mice were divided into the control (C, *n* = 15), ASC-EV (E, *n* = 15), and CM-EV (CM, *n* = 15) groups. Mice in the C, E, and CM groups were intravenously administered saline, ASC-EV, and CM-EV, respectively, once weekly from 6 to 42 weeks of age.

**Results:**

Compared to the ASC-EV, the CM-EV showed a significant increase in TGF-β1 production and miR-155-5p and miR-142-3p expression. CM-EV treatment increased survival, decreased anti-dsDNA antibody levels, and ameliorated renal histopathology. Although ASC-EV treatment significantly reduced the incidence of severe proteinuria and improved renal histopathology, it did not significantly improve survival rate.

ASC-EV or CM-EV treatment significantly decreased the proportion of pro-inflammatory macrophages (CD11c + CD206-; M1) and M1:M2 ratio. Additionally, CM-EV treatment significantly increased the expression of anti-inflammatory macrophages (CD11c-CD206 + ; M2). Moreover, CM-EV treatment significantly decreased the expression of lupus-specific miRNAs (miR-182-5p and miR-183-5p) in the spleen.

**Conclusions:**

EV derived from iMSCs primed with conditioned media obtained from disease-conditioned immune cells exert immunomodulatory effects and ameliorate SLE in a murine model.

**Supplementary Information:**

The online version contains supplementary material available at 10.1186/s13075-024-03435-1.

## Background

Systemic lupus erythematosus (SLE) is an incurable chronic autoimmune disease of unknown cause [1]. However, a combination of genetic, epigenetic, environmental, and hormonal factors and dysfunctional immune regulation are believed to contribute to SLE development [1]. The NZB/W F1 female mouse is an established SLE animal model that exhibits a spontaneous autoimmune disease process resembling that of human SLE; the NZB/W F1 female mouse produces anti-nuclear antibodies at 4–5 weeks of age; develops an immune complex-mediated glomerulonephritis at 4–5 months; and dies of renal failure by 10–12 months of age [2].

Current treatments for SLE include immunosuppressive drugs, corticosteroids, and antimalarials [3]; however, these drugs show various side effects with long-term use and are ineffective in some patients.

Mesenchymal stem cells (MSC) are emerging as a novel treatment strategy for several diseases, including difficult-to-treat autoimmune diseases, owing to their immunomodulatory, tissue regeneration, and protective effects [4]. Notably, the effectiveness of MSC treatment has been reported in animal models of various autoimmune diseases, such as rheumatoid arthritis, autoimmune encephalomyelitis, and SLE [5–7]. In a human clinical trial, MSC treatment significantly decreased SLE disease activity index (SLEDAI) and ameliorated proteinuria in patients with SLE compared to those in the control group [8]. Additionally, the administration of allogeneic MSCs to patients with SLE who were unresponsive to current medications resulted in clinical remission [9].

However, a disadvantage of MSC therapy is that it is difficult to continuously obtain cells with a stable phenotype from a consistent source. Moreover, the clinical application of MSC therapy is hindered by the loss of stemness during continuous culture and infusion-related toxicities, such as cell trapping in the lung microvasculature or myocardial microinfarction by intra-arterial infusion [10].

MSC-derived extracellular vesicles (EV) are small membrane-derived particles and contain several cellular constituents, such as mRNA, miRNA, cytokines, and growth factors. Thus, MSC-derived EV possess therapeutic potential similar to that of MSCs and do not cause side effects, such as immunogenicity and infusion toxicities [11]. Although MSC-derived EV therapy exerts immunomodulatory effects in several animal models of autoimmune diseases [12, 13], studies are yet to investigate the effects of long-term MSC-derived EV treatment in chronic autoimmune diseases.

MSCs undergo senescence as the passage number increases during prolonged in vitro culture [14], and thus it is essential to use low-passage MSCs to ensure optimal therapeutic effect. In contrast, immortalized MSCs (iMSCs) maintain their stemness and exhibit consistent characteristics, even during continuous culture, making them suitable for EV therapy studies, as they allow for reliable and consistent results [15].

The interactions between MSCs and the host microenvironment regulate the secretion of various factors by MSCs [16]. However, MSC-derived EV therapy did not change in response to the host microenvironment. We thus hypothesized that when MSCs are co-cultured with conditioned media containing various soluble factors (cytokines, EVs, etc.) derived from immune cells (splenocytes) from SLE-progressed mice, they are exposed to a microenvironment similar to that of the disease. This exposure could stimulate the MSCs to secrete more disease-relevant factors, potentially leading to the production of EV that are more effective in treating SLE.

Therefore, this study aimed to investigate the therapeutic effects of EV derived from iMSCs (ASC-EV) and iMSCs primed with conditioned media obtained from disease-conditioned immune cells (CM-EV) in a murine model of SLE (NZB/W F1 female mice). To the best of our knowledge, this is the first study to monitor the long-term effects of MSC-derived EV in a spontaneously induced SLE model.

## Methods

### iMSC culture, immunophenotype characteristics, and differentiation capacity

The immortalized adipose-derived MSC line used in this study was purchased from the ATCC (cat. No. SCRC-4000). iMSCs were cultured according to the manufacturer’s instructions [17]. The appropriate institutional review board (IRB) evaluated and approved this study (KWNUIRB-2021–04-009–002). The immunophenotypic characteristics and differentiation capacity of the iMSCs were determined as previously described [17].

### Preparation of splenocytes from old NZB/W F1 mice

Thirty-seven female NZB/W F1 mice (20-week-old) were purchased from the Jackson Laboratory (Bar Harbor, ME, USA). The spleen was collected from an old NZB/W F1 mouse (31–46 weeks old) that was judged have glomerulonephritis based on proteinuria and weight loss (active SLE). Splenocytes from each mouse were stored in liquid nitrogen (90% fetal bovine serum (FBS) and 10% dimethyl Sulfoxide) until further use. Splenocytes from each old mouse were used to prepare the conditioned media. The Institutional Animal Care and Use Committee (IACUC) of Kangwon National University approved this animal experiment (KW-211020–1). All procedures were performed in compliance with the Animal Welfare Act Regulations and Guide for the Care and Use of Laboratory Animals.

### Production and characterization of EV derived from the supernatant of iMSCs (ASC-EV) or iMSCs primed with conditioned media (CM-EV)

ASC-EV were produced as described previously [17]. CM-EV were produced as follows: cryopreserved splenocytes from old mice were seeded in Dulbecco’s modified Eagle’s medium (DMEM) complete medium (DMEM supplemented with 2 mmol/mL glutamine, 100 μg/mL penicillin/streptomycin, and 10% fetal bovine serum) for 24 h. The viability of thawed splenocytes, stained with trypan blue and measured using the LUNA II automated cell counter (Logos Biosystems, Anyang, South Korea), was 85.1 ± 1.7% (82.1–88.1%). The culture supernatant was centrifuged at 2000 × g for 30 min and filtered through a 0.2-μm filter (conditioned media). iMSCs were seeded in conditioned media for 24 h (10^7^ iMSCs per 175 T flask), followed by incubation in DMEM complete medium containing 10% exosome-depleted FBS (Gibco, Grand Island, NY, USA) for 48 h (CM-MSC).

ASC-EV or CM-EV were isolated using Total Exosome Isolation Reagent as described in our previous study [17]. The total protein concentration of ASC-EV or CM-EV was determined using bicinchoninic acid (BCA) assay. The size distribution of EV was analyzed using nanoparticle tracking analysis (NTA, Malvern Instruments Ltd., Worcestershire, UK), and CD81 (exosome marker) expression was measured using an ExoELISA-ULTRA Complete Kit (SBI, Palo Alto, CA, USA) according to the manufacturer’s instructions [17].

### Determination of the contents of transforming growth factor (TGF)-β1, interleukin (IL)-1Ra, and PGE2 in ASC-EV and CM-EV using ELISA

The contents of TGF-β1, IL-1Ra, and PGE2 in ASC-EV and CM-EV were determined using human LAP (TGF-β1) Quantikine ELISA Kit, human IL-1ra/IL-1F3 Quantikine ELISA Kit, and Prostaglandin E2 (PGE2) Parameter Assay Kit (R&D systems, Minneapolis, MN, USA), respectively [17].

### MicroRNA (miR)-10-5p, miR-142-3p, miR-146a-5p, miR-155-5p, and miR-216a-5p expression in iMSCs and EV

The expression levels of miR-10-5p, miR-142-3p, miR-146a-5p, miR-155-5p, and miR-216a-5p in iMSCs and EV were analyzed as previously described [17].

### Experimental animals

Forty-five female NZB/W F1 mice (4-week-old) were purchased from Jackson Laboratory and acclimatized for 2 weeks prior to the experiment. Mice were housed in a specific pathogen-free (SPF) house in GenNBio and had ad libitum access to food and water during the experimental period. Mice were divided into three groups: control (C, *n* = 15), ASC-EV (E, *n* = 15), and CM-EV (CM, *n* = 15). The number of animals per group were determined based on previous reports [7, 18]. Mice in the C group were intravenously injected via the tail vain with 150 μL of saline, whereas those in the E group were intravenously injected via the tail vain with EV derived from the supernatant of 1.33 × 10^6^ iMSCs (ASC-EV)/150 μL of saline. Mice in the CM group were intravenously injected via the tail vain with EV derived from the supernatant of 1.33 × 10^6^ iMSCs primed with conditioned media (CM-EV)/150 μL of saline once a week from age 6 weeks until age 42 weeks. This animal study was approved (GN-IACUC-22–05-07) by the IACUC of GenNBio. All procedures were performed in compliance with the Animal Welfare Act Regulations and Guide for the Care and Use of Laboratory Animals.

### Survival rate and incidence of severe proteinuria

Survival was monitored until 43 weeks of age, and the incidence of severe proteinuria (percentage of mice with urine protein ≥ 300 mg/dL) was determined until 42 weeks of age.

### Determination of anti-dsDNA antibody levels, proteinuria, and blood urea nitrogen (BUN)

After anesthetization using isoflurane, blood samples were collected from NZB/W F1 mice at 24, 32, 36, and 40 weeks of age and at autopsy (43 weeks of age). Serum samples were collected after centrifugation and stored at ˗80 °C until analysis. The serum levels of anti-dsDNA antibody were analyzed using mouse anti-dsDNA ELISA kits (FUJIFILM Wako Shibayagi Co. Shibukawa, Japan) [18]. Urine was collected fresh by means of abdominal massage. Urine protein concentration was measured at 2-week intervals using the Coomassie Brilliant Blue method, as described previously [18]. BUN levels were measured using FUJI-DRI-CHEM (NX500i, Fujifilm).

### Histopathological examination and immunofluorescence analysis of the kidneys

Histopathological examination and immunofluorescence analysis of the kidneys were performed as previously described [18]. Histopathological changes in the kidneys and the degree of IgG infiltration and C3 deposit in the glomerulus were graded from 0 (none) to 4 (severe) points; inflammatory cell infiltration, mesangial proliferation, tubular dilation, and fibrosis were scored on a 4-point scale as 0 (none), 1 + (mild), 2 + (moderate), 3 + (moderate to severe), and 4 + (severe).

### Flow cytometry

At the end of the animal experiment, mice were anesthetized using isoflurane (5% induction and 2% maintenance) and sacrificed by cervical dislocation. Splenocytes were obtained from mice, and flow cytometry was performed to analyze the proportion of T cells, macrophages, and T helper cell subsets in the spleen, as previously described [19, 20]. These experiments are described in detail in the supplementary information.

### Determination of spleen lupus-specific miRNA expression

miRNAs were isolated from the spleen using a miRNA mini kit (Qiagen). cDNA synthesis and qPCR were performed as described previously [21]; the relative expression of the target miRNAs (miR-31-5p, miR-96-5p, miR-127-3p, miR-155-5p, miR-182-5p, miR-183-5p, and miR-379-5p) was calculated using the 2^−ΔΔCT^ method with U6 snRNA as the housekeeping gene [21].

### Measurement of serum cytokine levels

Serum levels of various cytokines were measured using Milliplex® MAP Kits (Millipore, Burlington, Massachusetts, USA) as previously described [19, 20].

### Terminal deoxynucleotidyl transferase dUTP nick-end labeling (TUNEL) assay of renal tubular epithelial cells

TUNEL assay was used to assess the degree of apoptosis in renal tubular epithelial cells using a TUNEL Assay Kit (Fluorescence, 488 nm, Cell Signaling Technology), according to the manufacturer’s instructions.

### Statistical analysis

All data are presented as mean ± standard error of mean (SEM), with the exception of survival rate and incidence of severe proteinuria. The survival rate and incidence of severe proteinuria were compared among the groups using Kaplan–Meier curves and the log-rank test. Anti-dsDNA antibody levels and serum cytokine levels were compared among groups using the Kruskal–Wallis test and the Dunn’s test, while other data were compared among groups using a one-way analysis of variance (†: *p* < 0.05), followed by post-hoc Tukey’s multiple-comparison tests (*: *p* < 0.05). Statistical significance was set at *p* < 0.05. All statistical analyses were conducted using SPSS version 26.0 (IBM, Armonk, NY, USA).

## Results

### iMSC immunophenotyping and differentiation

**Fig. 1:**
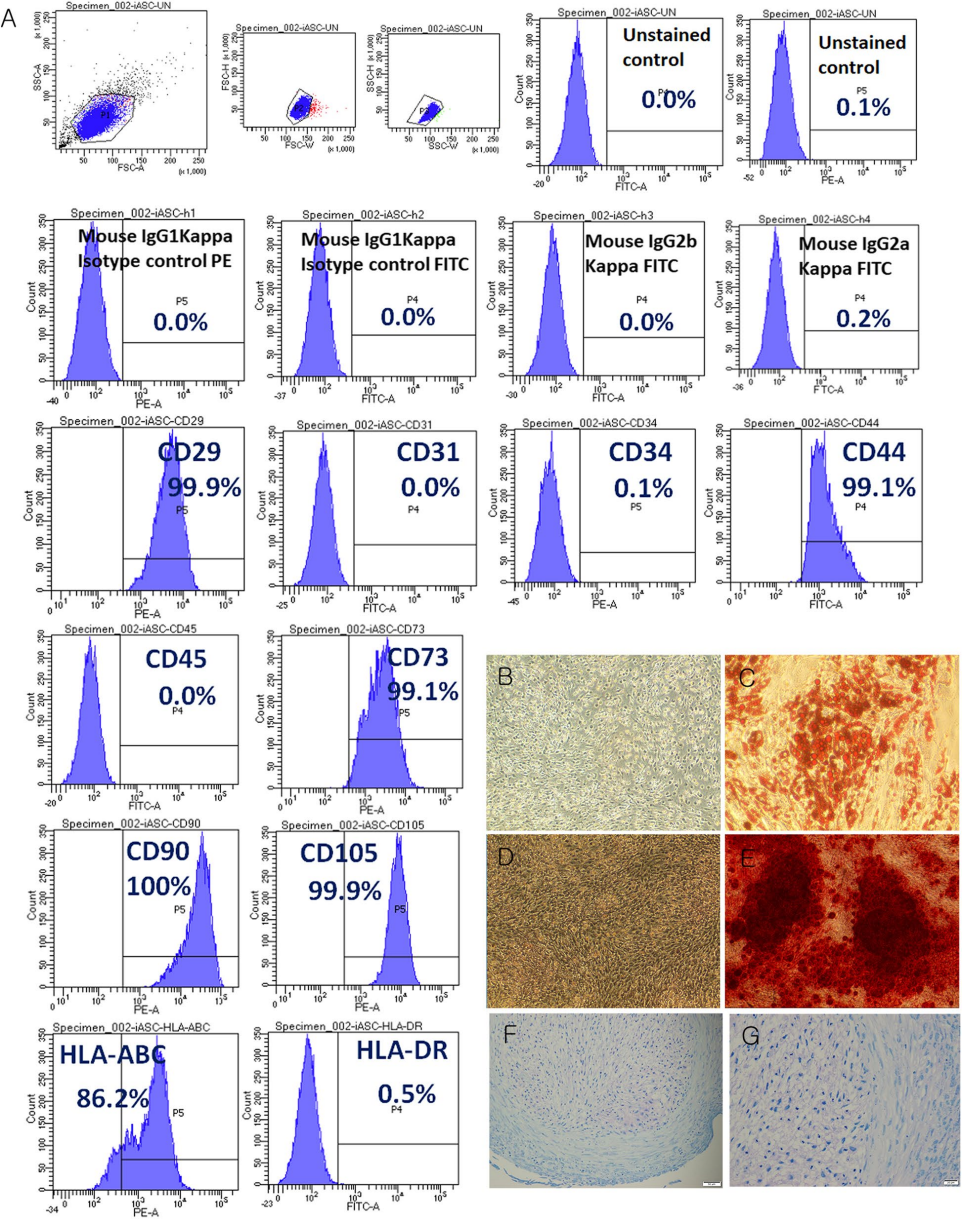
Immunophenotype and differentiation capacity of immortalized mesenchymal stem cells (iMSCs). **A** Immunophenotype of iMSCs used in this study. **B** Negative control of iMSCs; Oil Red-O staining, magnification = × 100. **C** Lipid droplets were observed in adipocytes differentiated from iMSCs; Oil Red-O staining, magnification = × 100. **D** Negative control of iMSCs; Alizarin red staining, magnification = × 100. **E** Osteocytes differentiated from iMSCs; Alizarin red staining, magnification = × 100; Scale bar: 100 μm. **F** Aggregated chondrocytes differentiated from iMSCs were stained with toluidine blue; magnification = × 200; Scale bar: 50 μm **G** Aggregated chondrocytes derived from iMSCs were stained with toluidine blue; magnification = × 400; Scale bar: 20 μm

The results of analysis of immunophenotypic characteristics and differentiation potential of the iMSCs used herein are shown in Fig. 1. iMSCs strongly expressed CD29, CD44, CD73, CD90, CD105, and HLA-ABC, while being negative for CD31, CD34, and HLA-DR expression. Upon receiving the appropriate differentiation stimuli, they differentiated into adipocytes, osteocytes, and chondrocytes. iMSCs primed with conditioned media (CM-MSC) also strongly expressed CD29, CD44, CD73, CD90, CD105, and HLA-ABC, while being negative for CD31, CD34, and HLA-DR expression (Supplementary Fig. 1).

### EV characterization

**Fig. 2:**
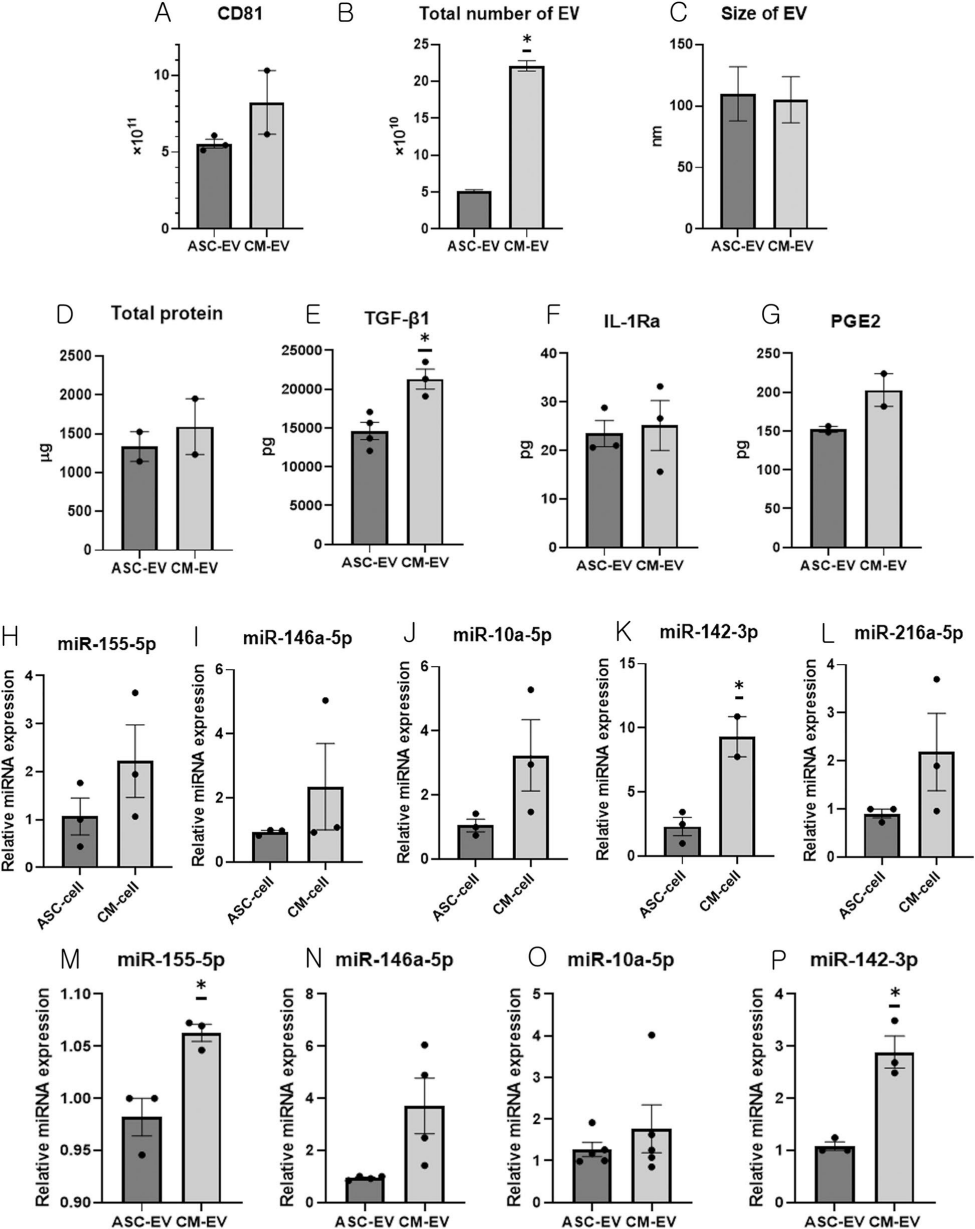
Characterization of extracellular vesicles (EV). **A** Total CD81 expression of EV produced from 10^7^ iMSCs (ASC-EV) or iMSCs primed with conditioned media obtained from disease-conditioned immune cells (CM-EV). **B** Total number of EV obtained from 10^7^ iMSCs or CM-MSC was measured using NTA (*n* = 5). **C** Sizes of ASC-EV and CM-EV were measured using NTA (*n* = 5). **D** Total protein contents of ASC-EV and CM-EV produced from 10^7^ iMSCs or CM-MSC. **E** Amounts of TGF-β1 in ASC-EV and CM-EV produced from 10^7^ iMSCs or CM-MSC. **F** Amounts of IL-1Ra in ASC-EV and CM-EV produced from 10^7^ iMSCs or CM-MSC. **G** Amounts of PGE2 in ASC-EV and CM-EV produced from 10^7^ iMSCs or CM-MSC. Relative expression of **H** miR-155-5p, **I** miR-146-5p, **J** miR-10-5p, **K** miR-142-3p, and **L** miR-216a-5p in iMSCs or CM-MSC. Relative expression of **M** miR-155-5p, **N** miR-146-5p, **O** miR-10-5p, and **P** miR-142-3p in ASC-EV and CM-EV. Data (mean ± standard error of mean [SEM]) were compared using Student’s *t*-test. *, Significantly different (*p* < 0.05) compared with ASC-EV. Sample number: miRNA (H–P: *n* = 3–5/group, duplicate), iMSCs: immortalized mesenchymal stem cells, ASC-EV: EV derived from the supernatant of iMSCs, CM-EV: EV derived from the supernatant of iMSCs primed with conditioned media obtained from disease-conditioned immune cells, CM-MSC: iMSCs primed with conditioned media obtained from disease-conditioned immune cells, NTA: nanoparticle tracking analysis, miR: microRNA

The total amount of CD81 in ASC-EV produced from the supernatant of 10^7^ iMSCs and CM-EV produced from the supernatant of 10^7^ iMSCs primed with conditioned media (CM-MSC) is presented in Fig. 2A. NTA revealed that the total number of ASC-EV produced from the supernatant of 10^7^ iMSCs was 5.20 × 10^10^ ± 0.15 × 10^10^, whereas that of CM-EV produced from the supernatant of 10^7^ iMSCs primed with conditioned media was 22.1 × 10^10^± 0.7 × 10^10^ (Fig. 2B). Notably, the total number of EV was significantly higher (Student’s t-test, *p* < 0.001) in the CM-EV group than in the ASC-EV group. Moreover, the average sizes of ASC-EV and CM-EV were 109.8 ± 22.0 nm and 105.1 ± 18.7 nm, respectively (Fig. 2C).

### Contents of TGF-β1, IL-1Ra, and PGE2 in EV

Analysis of EV produced from 10^7^ iMSCs or CM-MSC revealed that the amount of total protein was 1043.8 ± 100.8 μg in ASC-EV, and 1593.5 ± 359.8 μg in CM-EV (Fig. 2D).

Analysis of EV produced from 10^7^ iMSCs or CM-MSC revealed that the amount of TGF-β1 was 14,615.5 ± 1101.4 pg in ASC-EV and 21,312.87 ± 1278.4 pg in CM-EV; the amount of TGF-β1 in CM-EV was significantly higher than that in ASC-EV (Student’s t-test, *p* = 0.011; Fig. 2E). Analysis of EV produced from 10^7^ iMSCs or CM-MSC revealed that the amount of IL-1Ra was 23.5 ± 2.7 pg in ASC-EV and 25.1 ± 5.1 pg in CM-EV (Fig. 2F). The amount of PGE2 was found to be 152.3 ± 3.9 pg in ASC-EV and 202.9 ± 21.2 pg in CM-EV (Fig. 2G).

### Expression of miR-10-5p, miR-142-3p, miR-146a-5p, miR-155-5p, and miR-216a-5p in iMSCs and EV

Quantitative real-time polymerase chain reaction (qRT-PCR) showed that miR-142-3p expression was significantly higher (Student’s t-test, *p* = 0.018) in CM-MSC than in iMSCs (Figs. 2H–L). Similarly, miR-155-5p (Student’s t-test, *p* = 0.015) and miR-142-3p (Student’s t-test, *p* = 0.005) expression levels were significantly higher in CM-EV than in ASC-EV (Figs. 2M–P).

### Survival rates and incidence of severe proteinuria

**Fig. 3:**
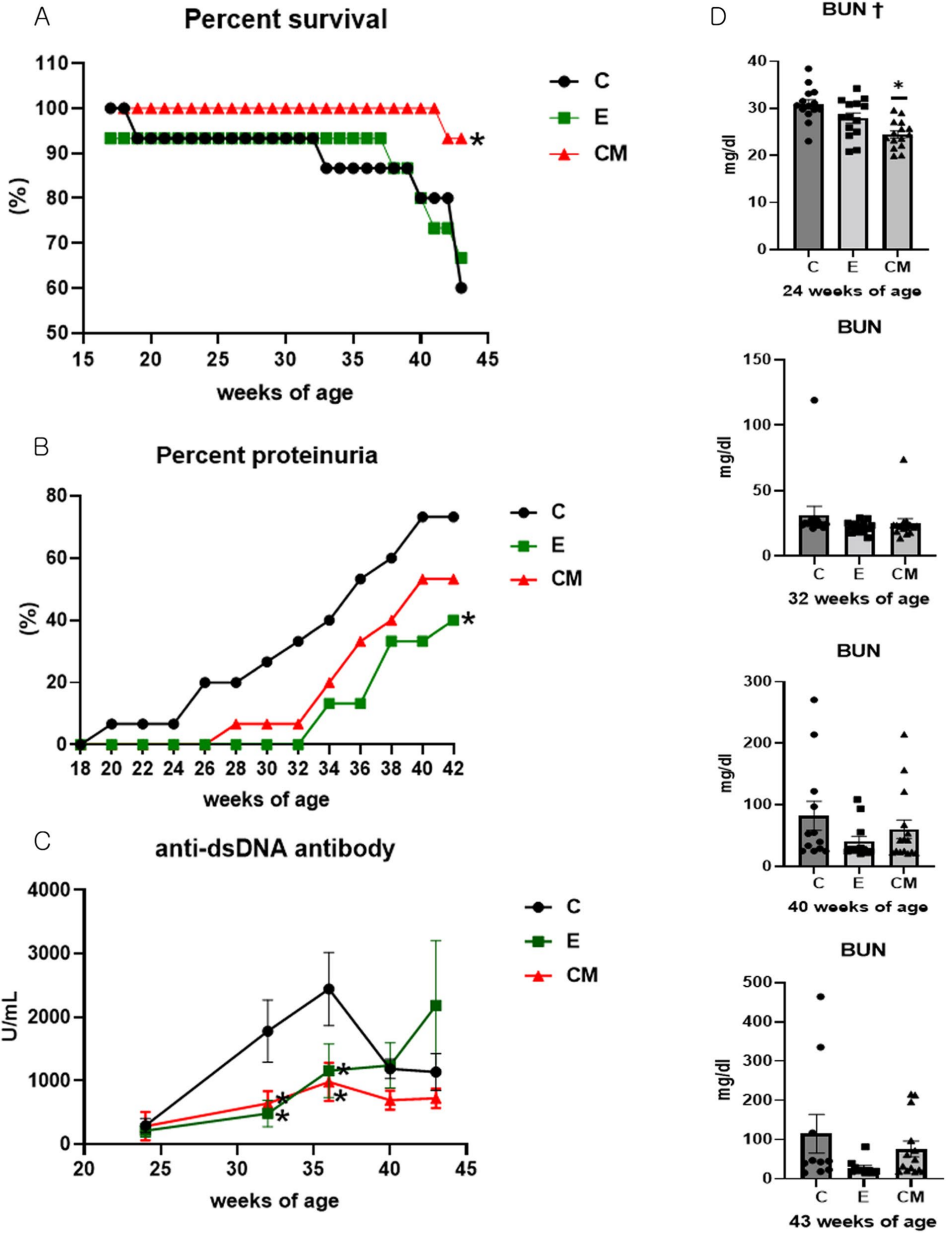
Survival rate, incidence of severe proteinuria, serum levels of anti-dsDNA antibody, and blood urea nitrogen (BUN) concentration. Survival was monitored until 43 weeks of age, and the incidence of severe proteinuria (percentage of mice with urine protein ≥ 300 mg/dL) was determined until 42 weeks of age (*n* = 15 per group). **A** Survival rate, **B** Percentage of incidence of severe proteinuria, **C** Serum levels of anti-dsDNA antibodies, **D** BUN concentration. Data from (A) and (B) were compared using Kaplan–Meier curves and the log-rank test, Data from (C) were compared using the Kruskal–Wallis and Dunn’s tests, while data (mean ± SEM) from (D) were compared using a one-way analysis of variance (†: *p* < 0.05) followed by Tukey’s post-hoc tests. *: Significantly different compared with the control group (vs. C groups, *p* < 0.05). Sample number: all samples from live animals per group Sample number: all samples from live animals per group (1–16w: C = 15, E = 14, CM = 15, 17–18w: C = 15, E = 14, CM = 15, 19–32w: C = 14, E = 14, CM = 15, 33–37w: C = 13, E = 14, CM = 15, 38–39w: C = 13, E = 13, CM = 15, 40w: C = 12, E = 12, CM = 15, 41–42w: C = 12, E = 11, CM = 15, 43w: C = 10, E = 10, CM = 14), C: control (dPBS treatment group), E: ASC-EV treatment group, CM: CM-EV treatment group, w: weeks of age

One mouse in the C group that was moribund was included as deceased. The survival rate at 43 weeks of age was 60%, 66.7%, and 93.3% in groups C, E, and CM, respectively. The survival rate was significantly higher in the CM group than in the C group (Log-rank test, *p* = 0.111; pairwise comparison, C vs. CM: *p* = 0.036, Fig. 3A). Moreover, compared to the C group, the E group had a significantly decreased incidence of severe proteinuria (Log-rank test, *p* = 0.105; pairwise comparison, C vs. E, *p* = 0.046, Fig. 3B).

### Anti-dsDNA antibody and BUN levels

Serum anti-dsDNA antibody concentrations at 32 and 36 weeks of age differed significantly among the groups (Kruskal–Wallis, *p* = 0.007 and *p* = 0.027, respectively). Notably, serum anti-dsDNA antibody levels were significantly lower in the E and CM groups than in the C group at 32 (Dunn’s test; C vs. E: *p* = 0.002, C vs. CM: *p* = 0.029) and 36 weeks of age (C vs. E: *p* = 0.016; C vs. CM: *p* = 0.023; Fig. 3C). Additionally, BUN levels were significantly lower (ANOVA & Tukey’s test, *p* < 0.001) in the CM group than in the C group at 24 weeks of age (Fig. 3D).

### Histopathological examination of the kidneys

**Fig. 4:**
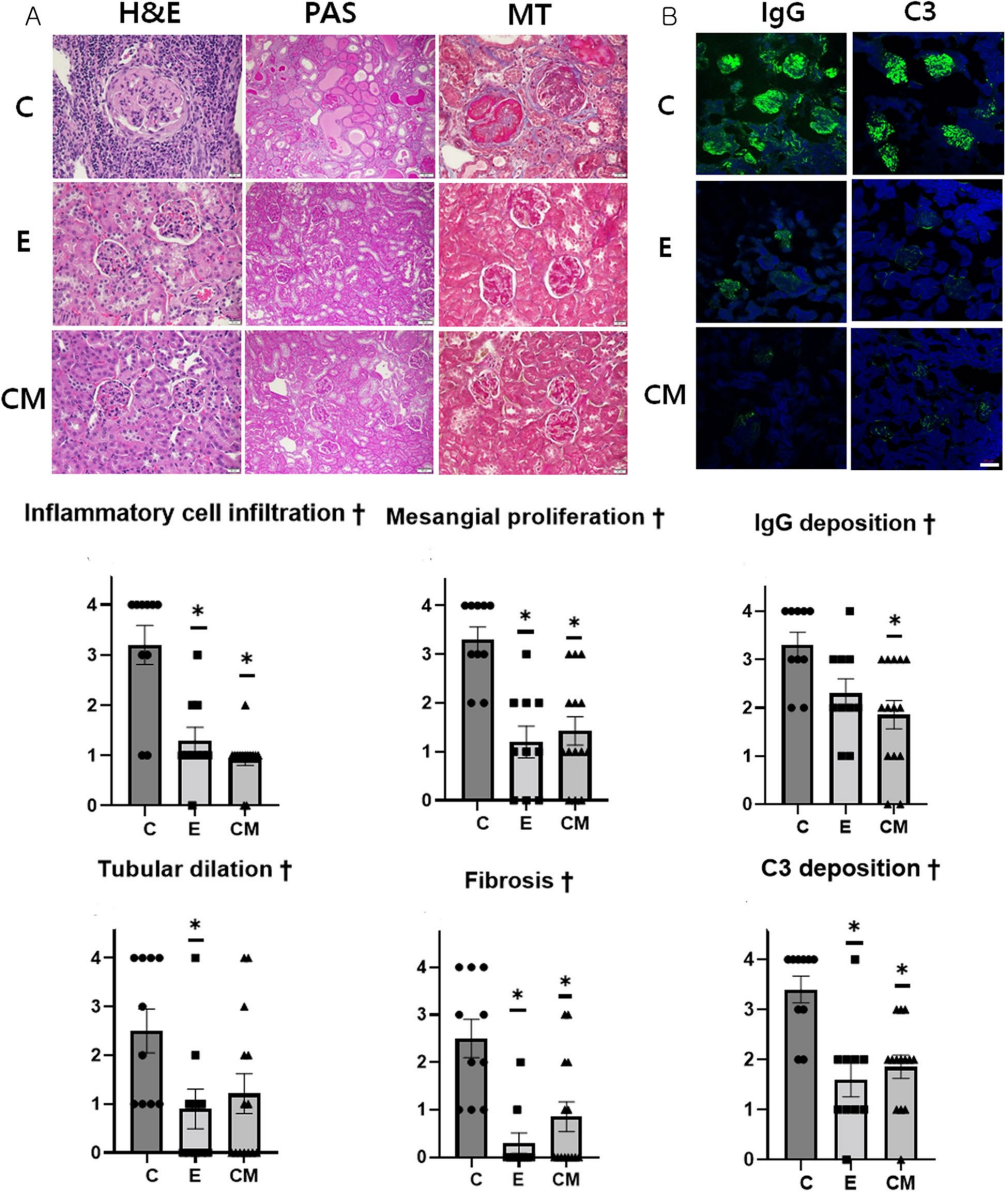
Histopathological and immunofluorescent examinations of kidneys at the end of the experiment. **A** Hematoxylin and eosin (H&E, [× 400, scale bar: 20 μm]), periodic acid-Schiff (PAS; [× 200, scale bar: 50 μm]), and Masson’s trichrome staining (× 400, scale bar: 20 μm) of kidneys collected from mice at 43 weeks of age, and histopathological scores (mean ± standard error of mean [SEM]). **B** IgG and C3 deposition in kidneys (× 200, scale bar: 50 μm), and the score of fluorescence staining intensities of IgG and C3 deposits (mean ± SEM). Intergroup analysis was performed using a one-way analysis of variance (†: *p* < 0.05), followed by Tukey’s post-hoc tests. *: Significantly different compared with the control group (vs. group C, *p* < 0.05). Sample number: all samples at the end of study (C = 10, E = 10, CM = 14), C: control (dPBS treatment group), E: ASC-EV treatment group and CM: CM-EV treatment group

Histopathological evaluation of the kidneys showed obvious inflammatory cell infiltration, mesangial cell proliferation, tubule dilation, and fibrosis in the C group. However, the degree of inflammatory cell infiltration, mesangial cell proliferation, and fibrosis was significantly lower (ANOVA &Tukey’s test: *p* < 0.001) in the E and CM groups than in the C group (Fig. 4A). Moreover, the degree of tubular dilation was significantly lower (ANOVA &Tukey’s test: *p* = 0.04) in the E group than in the C group (Fig. 4A).

### IgG infiltration and C3 deposition in the kidneys

Glomerular IgG infiltration was significantly lower (ANOVA &Tukey’s test: *p* = 0.005) in the CM group than in the C group (Fig. 4B). Additionally, the degree of C3 deposition in the glomeruli was significantly lower (ANOVA &Tukey’s test: *p* < 0.001) in the E and CM groups than in the C group (Fig. 4B).

### Flow cytometry

**Fig. 5:**
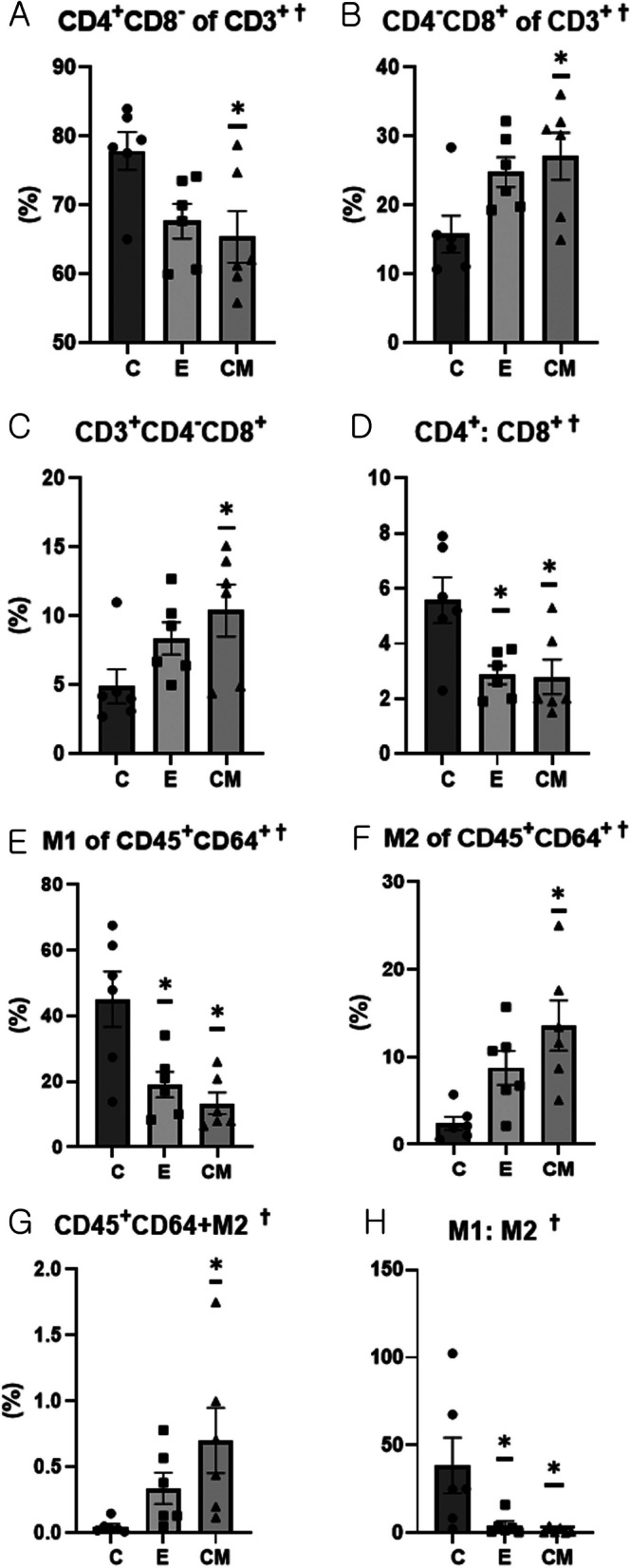
The proportions of T cell subsets and macrophages in the spleen determined by flow cytometry. The proportions of CD4^+^CD8^−^ and CD4^−^CD8^+^ among CD3^+^ cells and CD3^+^CD4-CD8^+^ cells and the CD4^+^CD8^−^:CD4^−^CD8^+^ ratio. The proportions of CD11c^+^CD206^−^ and CD11c^−^CD206^+^ cells among CD45^+^CD64^+^ cells; M2 macrophages (CD45^+^CD64^+^CD11c^−^CD206^+^); and M1:M2 ratio in the spleen (*n* = 6 per group). Data (mean ± standard error of mean [SEM]) were compared using a one-way analysis of variance (†: *p* < 0.05) followed by Tukey’s post-hoc tests. *: Significantly different compared with the control group (vs. group C, *p* < 0.05). C: control (dPBS treatment group), E: ASC-EV treatment group and CM: CM-EV treatment group

The proportion of CD4^+^CD8^-^ cells among the CD3^+^ cells was significantly lower in the CM group than in the C group (ANOVA &Tukey’s test, *p* = 0.025, Fig. 5A). Additionally, the proportions of CD4^-^CD8^+^ cells among CD3^+^ cells (ANOVA &Tukey’s test, *p* = 0.029) and CD3^+^ CD4^-^CD8^+^ cells (ANOVA &Tukey’s test, *p* = 0.054) were higher in the CM group than in the C group (Figs. 5B and C). Moreover, the CD4^+^ :CD8^+^ ratio was lower in the E and CM groups than in the C group (ANOVA &Tukey’s test, *p* = 0.054, Fig. 5D). Furthermore, the proportion of CD11c^+^ CD206^- ^macrophages (M1) among CD45^+^ CD64 ^+^ cells was significantly lower in the E and CM groups than in the C group (ANOVA &Tukey’s test, *p* = 0.003, Fig. 5E). In contrast, the proportion of CD11c-CD206 + macrophages (M2) was significantly higher in the CM group than in the C group (ANOVA &Tukey’s test, *p* = 0.006, Fig. 5F). Additionally, the proportion of CD45^+^CD64^+^CD11c^-^CD206^+^ cells was significantly higher in the CM group than in the C group (ANOVA &Tukey’s test, *p* = 0.034, Fig. 5G). Moreover, the M1:M2 ratio was significantly lower in the E and CM groups than in the C group (ANOVA &Tukey’s test, *p* = 0.022, Fig. 5H). A representative gating scheme of the T cell subset is presented in Supplementary Fig. 2, while the macrophage subset is shown in Supplementary Fig. 3.

However, no significant differences were observed in the proportions of CD4 + CD25 + Foxp3 + (Treg), CD4 + CD25 + ROR-γt + (Th17), CD4 + CD25 + T-bet + (Th1), and CD4 + CD25 + GATA3 + (Th2) among the groups (Supplementary Fig. 4). 

### Lupus-specific miRNA expression in spleen

**Fig. 6:**
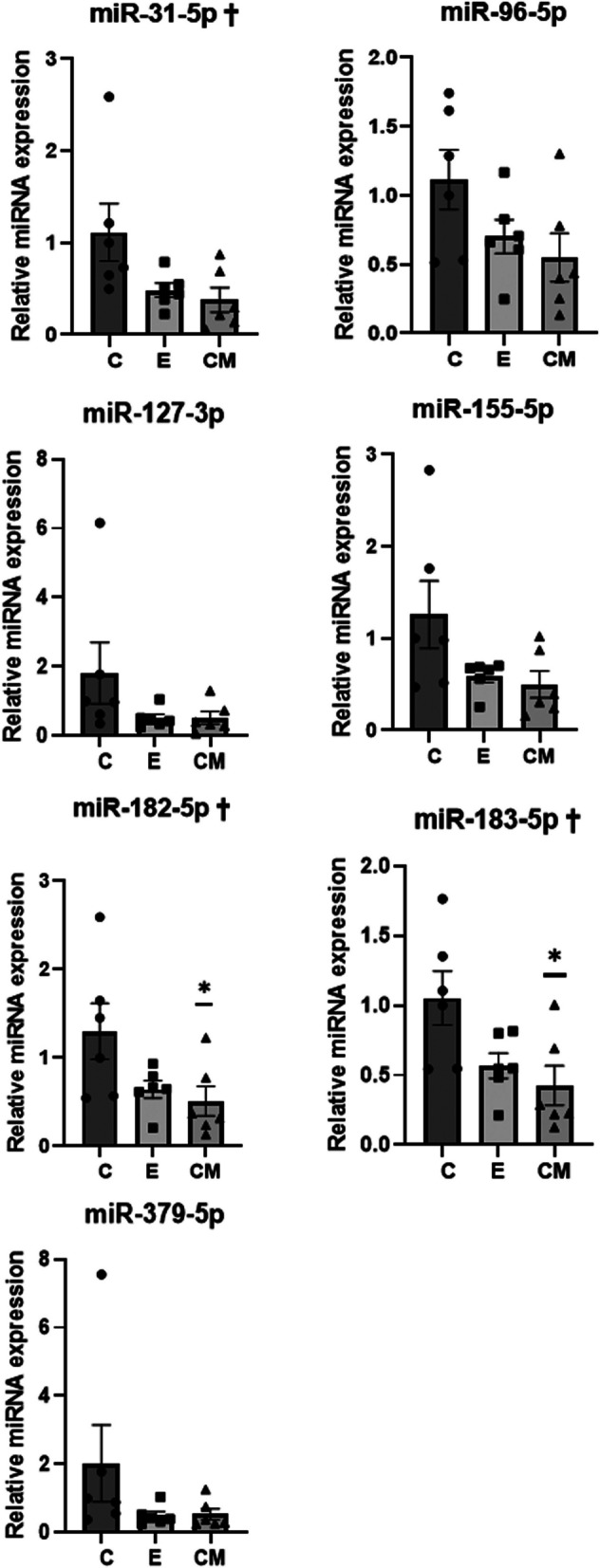
The expression levels of various common lupus disease-associated miRNAs in spleens. U6 snRNA served as the housekeeping gene, and the method of 2^−ΔΔCT^ was used to determine the relative expression levels of miRNAs. The expression levels of miRNA are expressed as mean ± SEM (bars ± error bars, *n* = 6 per group, duplicate) of the relative expression compared to one sample from the control group. The relative expression levels of miRNAs were compared using a one-way analysis of variance (†: *p* < 0.05) followed by post-hoc Tukey’s multiple comparison tests. *: Significant difference from the control group (vs. group C, *p* < 0.05). C: control (dPBS treatment group), E: ASC-EV treatment group and CM: CM-EV treatment group

qRT-PCR showed that there were significant differences in the expression of miR-31-5p, miR-182-5p, and miR-183-5p among the groups (ANOVA &Tukey’s test: *p* = 0.044, *p* = 0.044, and *p* = 0.023, Fig. 6). Specifically, the mean expression of all lupus-specific miRNAs was the highest in the C group, and the expression of miR-182-5p and miR-183-5p was significantly lower in the CM group than in the C group.

### Serum cytokine levels

**Fig. 7:**
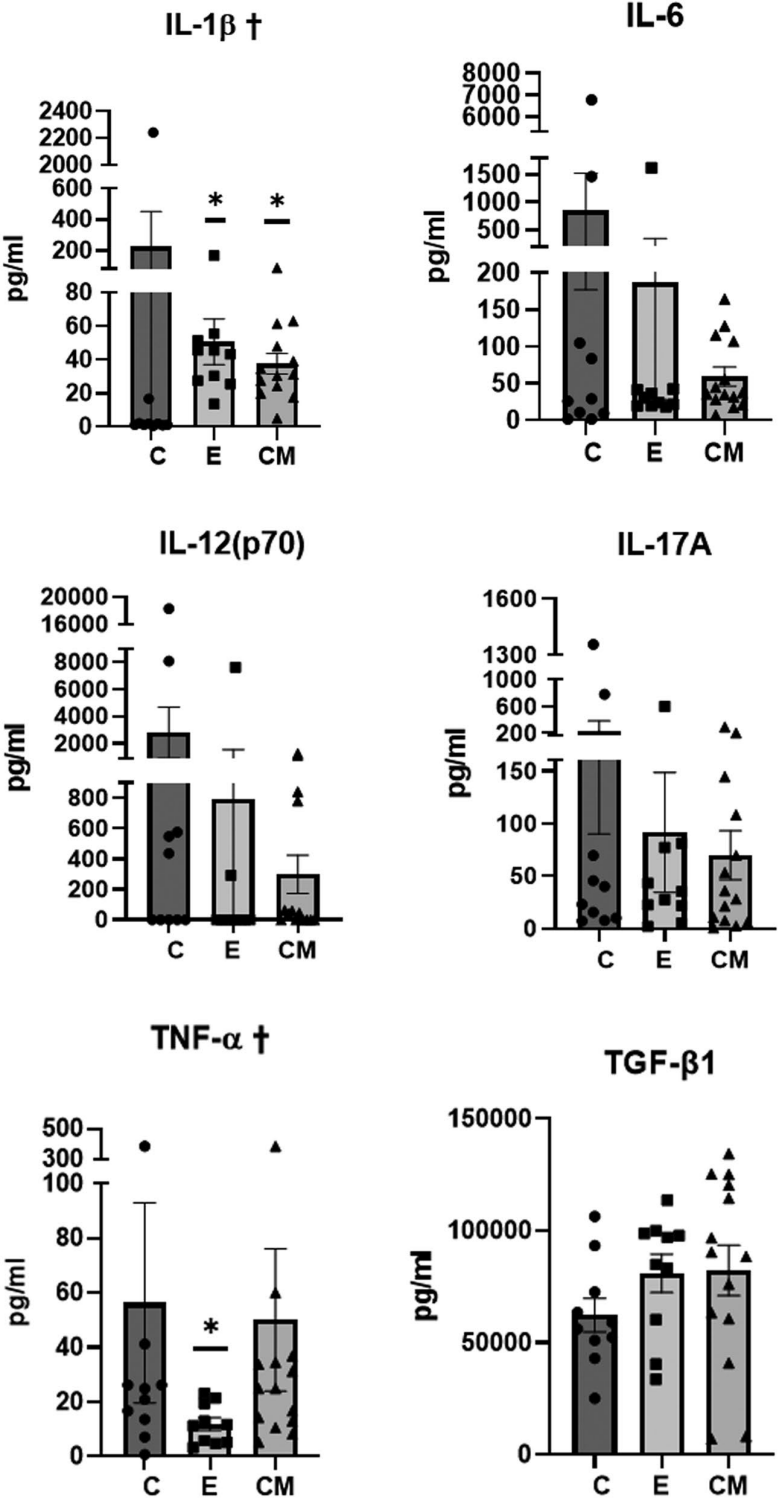
Serum levels of pro-inflammatory cytokines and TGF-β1. Data (mean ± standard error of mean [SEM]) were compared using the Kruskal–Wallis (†: *p* < 0.05) & Dunn’s tests. *: Significantly different compared with the control group (vs. group C, *p* < 0.05). Sample number: all samples at the end of study (C = 10, E = 10, and CM = 14), C: control (dPBS treatment group), E: ASC-EV treatment group and CM: CM-EV treatment group

The mean serum levels of IL-1β, IL-6, IL-12(p70), IL-17a, and TNF-α were the highest in the C group. The mean serum TGF-β1 level was the lowest in the C group compared with that in the other groups. Notably, there were significant differences in the serum levels of IL-1β (Kruskal–Wallis, *p* = 0.002) and TNF-α (Kruskal–Wallis, *p* = 0.039) among the groups; serum levels of IL-1β were significantly lower in the C group than in the E and CM groups (Dunn’s test, C vs E: *p* = 0.001 and C vs CM: *p* = 0.003). Serum levels of TNF-α were significantly lower in the E group than in the C group (Dunn’s test, C vs E: *p* = 0.048, Fig. 7).

### TUNEL assay

**Fig. 8:**
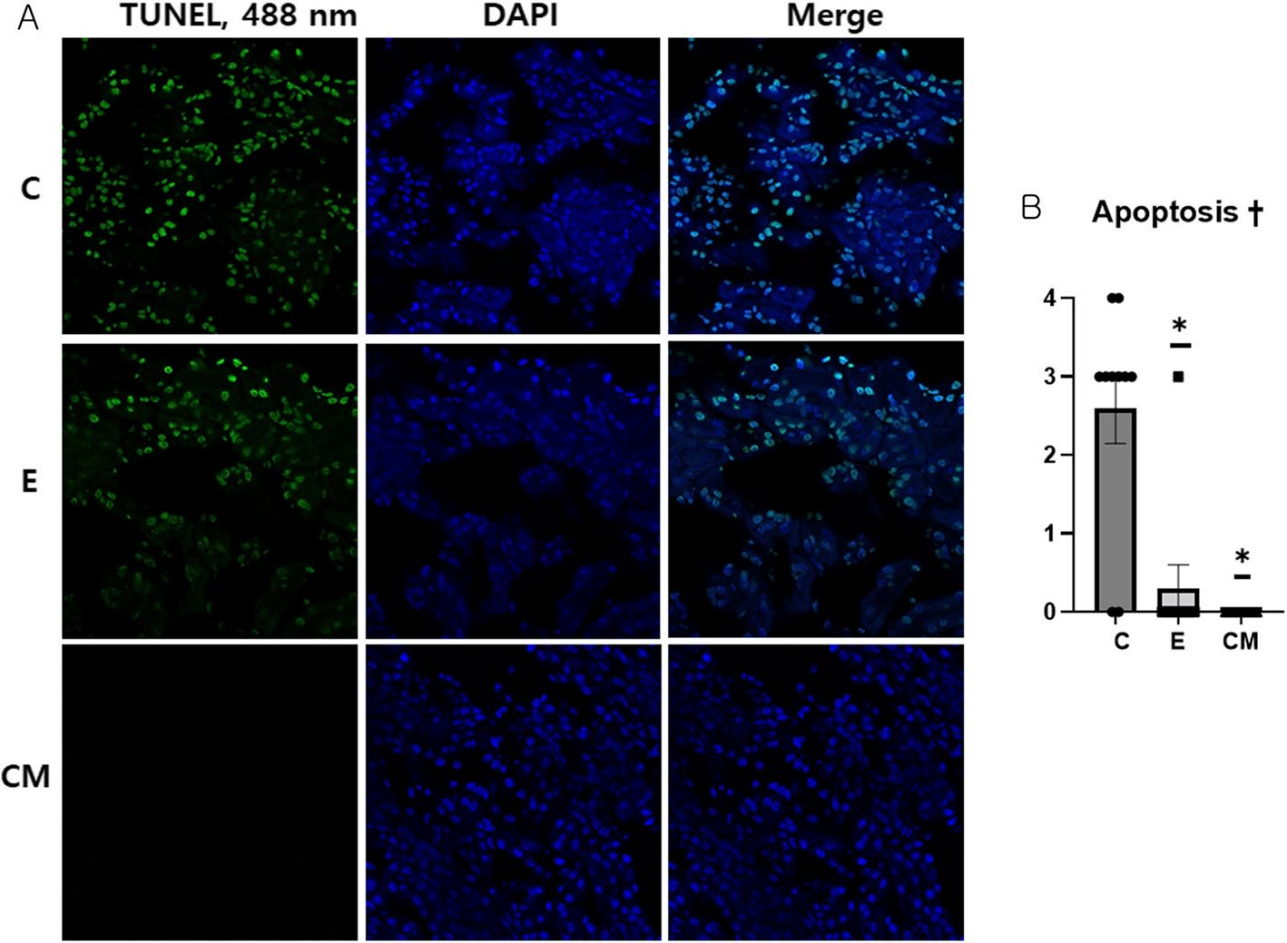
TUNEL assay to evaluate cell apoptosis in renal tubular epithelium. **A** TUNEL assay of kidneys collected from mice at 43 weeks of age. Representative kidney sections of each group. **B** The score of fluorescence staining intensities of TUNEL (apoptosis). The scores of fluorescence staining intensities of TUNEL (mean ± SEM) were analyzed using a one-way analysis of variance (†: *p* < 0.05) followed by Tukey’s post-hoc tests (*). *: Significantly different compared with the control group (vs. group C, *p* < 0.05). Sample number: all samples at the end of study (C = 10, E =10, and CM = 14), TUNEL: Terminal deoxynucleotidyl transferase dUTP nick-end labeling, C: control (dPBS treatment group), E: ASC-EV treatment group and CM: CM-EV treatment group

The rate of apoptosis of renal tubular epithelial cells was significantly higher in the C group than in the E and CM groups (ANOVA and Tukey’s test, *p* = 0.001; Figs. 8A and B).

## Discussion

Previous studies have demonstrated the effectiveness of MSC-EV in treating various autoimmune diseases using animal models. Reduction in clinical scores, demyelination, and neuroinflammation has been observed with MSC-EV therapy in the experimental autoimmune encephalomyelitis mouse model of multiple sclerosis, with an associated upregulation of Treg cells [22]. Additionally, MSC-EV treatment has been shown to decrease uveitis in experimental autoimmune uveitis rats [23]. Furthermore, MSC-EV therapy delayed the inflammatory response in rheumatoid arthritis by reducing cartilage damage and decreasing pro-inflammatory cytokines [12].

In the present study, we examined the therapeutic effects of CM-EV in SLE. CM-EV treatment significantly increased survival rates, decreased anti-dsDNA antibody levels, ameliorated renal histopathology and IgG and C3 deposition in the kidneys, and significantly decreased BUN levels at 24 weeks of age. Although ASC-EV significantly reduced the incidence of severe proteinuria, it did not improve survival rate.

Compared with those in ASC-EV, TGF-β1 production and expression of miR-155-5p and miR-142-3p in CM-EV significantly increased. NTA showed that CM-EV output from iMSC culture supernatant primed with conditioned media obtained from disease-conditioned immune cells was significantly higher than ASC-EV output from unprimed media.

In autoimmune diseases, miR-10-5p, miR-142-3p, miR-146a-5p, miR-155-5p, and miR-216a-5p contribute to the immunoregulatory effects of MSCs and EV derived from MSCs [24–26]. In this study, the expression of miR-155-5p and miR-142-3p was significantly higher in CM-EV than in ASC-EV. EV-derived miR-155-5p reduces B cell proliferation and PI3K/AKT-induced activation [24]. miR-142-3p expression in CD4 + T lymphocytes from patients with SLE is significantly lower than that of those from the healthy control group, resulting in T cell activation and B cell hyperstimulation [25].

An imbalance in the M1:M2 ratio and abnormal activation of macrophages contribute to the pathogenesis of SLE. MSCs can induce macrophage polarization toward the M2 phenotype through the TGF-β/Akt/FoxO1 pathway [27]. miRNA-155-5p in MSC-derived EV contributes to this mechanism [24]. Based on flow cytometry analysis of splenocytes, CM-EV treatment significantly increased the expression of anti-inflammatory M2 macrophages compared with that in the control group. Moreover, TGF-β is a major immunomodulating cytokines produced by MSCs. The activation of the TGF-β/NF-kB signaling pathway decreases the proportion of Th17 and Th1 and increases the proportion of Treg and Th2 [28]. In this study, the proportions of Treg, Th17, Th1, and Th2 cells were not significantly affected by the treatments. 

After disease onset, the expression of lupus-specific miRNAs, including miR-31, miR-96, miR-127, miR-155, miR-182, miR-183, and miR-379, was significantly upregulated in the splenocytes of NZB/W F1 mice [29]. Consistent with previous finding, lupus-specific miRNAs showed the highest expression in the splenocytes of NZB/W F1 mice in the C group but the lowest expression in the CM group. Particularly, the expression levels of miR-31-5p, miR-182-5p, and miR-183-5p were significantly different between the groups, with miR-182-5p and miR-183-5p being significantly downregulated in the CM group compared with that in the C group.

miR-31 targets forkhead-box p3 (Foxp3); therefore, miR-31 upregulation is associated with Treg dysfunction in lupus [30]. In SLE, miR-182–96-183 upregulation induces a decrease in Foxo1/3a and/or microphthalmia-associated transcription factor (MITF), which plays a role in regulating T cell homeostasis and tolerance, resulting in T and B cell activation, autoantibody production, immune tolerance breakdown, and autoimmunity development [31]. Therefore, decreased expression of the SLE disease-specific miRNAs (miR-31, miR-182, and miR-183) in the spleen may contribute to the alleviation of SLE.

Patients with active SLE have significantly higher serum levels of IL-1β and TNF-α than those with inactive SLE disease and healthy individuals [32]. In the present study, serum levels of IL-1β were significantly lower in the C group than in the E and CM groups, while serum levels of TNF-α were significantly lower in the E group than in the C group.

Furthermore, cell apoptosis was significantly higher in the renal tubular epithelium of mice in the C group than in that of those in the E and CM groups. Renal tubular epithelial cells affect the pathological process of acute and chronic kidney disease and play a major role in the incidence of lupus nephritis [33]. Therefore, suppressing apoptosis in the renal tubular epithelium may contribute to alleviating SLE.

In this study, there was no positive-treatment control group using current treatment drugs; however, the prednisolone treatment group (methylprednisolone 5 mg/kg/day) in our previous SLE study had a survival rate of 93.3% at 42 weeks of age [18]. Therefore, the survival rates of the CM-EV treatment group in this study and those of the prednisolone treatment group in the previous study were similar. CM-EV might be an alternate treatment if prednisolone is not working or if there are severe adverse effects.

There are obvious limitations in evaluating treatment effect in SLE mouse models [2, 19]; human SLE exhibits various clinical symptoms, such as nephritis, arthritis, and dermatitis. However, the NZB/W F1 mouse model of SLE is mainly characterized by the development of autoantibodies and lupus nephritis [2]. Therefore, although there are various evaluation criteria for human clinical symptoms, only the survival rate and the incidence of severe proteinuria were considered significant clinical markers in this experiment.

The amount of ASC-EV used in this experiment improved several indicators of SLE (decrease in the incidence of severe proteinuria and anti-dsDNA antibody concentration, reduction of proinflammatory macrophages, improvement of kidney histopathology, and reduction of apoptosis in renal tubular epithelial cells). However, this did not significantly increase survival rate. Since the therapeutic efficacy of EV is dose-dependent [34], it was expected that increasing the amount or frequency of administration of ASC-EV would show better effects in treating SLE.

Cytokine and histopathological examinations were performed on surviving individuals, revealing that the degree of kidney pathology was similar between the ASC-EV group and CM-EV group. The mice that died in the ASC-EV group showed severe proteinuria, indicating that they likely had a poor score in kidney pathology. Further research is needed to explore the mechanisms underlying the differences in survival rates between the ASC-EV and CM-EV groups. Further, herein, CM-EV showed a significant increase in TGF-β1 production and miR-155-5p and miR-142-3p expression compared to those in ASC-EV. The expression of TGF-β1, miR-155-5p, and miR-142-3p can significantly facilitate the treatment of autoimmune diseases [24–26]. Additionally, CM-EV treatment significantly reduced the expression of lupus-specific miRNAs (miR-182-5p and miR-183-5p) in the spleen, indicating that it was more effective than ASC-EV treatment in controlling SLE.

MSCs are a key source of various immunosuppressive soluble factors. One of the negative effects of MSC therapy or MSC-EV therapy is that the inclusion of many different materials makes it difficult to determine which specific components play the most significant role in disease suppression. In this study, we focused on TGF-β1, IL-1Ra, and PGE2, which are known to play the most important roles among the available factors known to contribute to immune regulation in autoimmune diseases [35], and measured their production. The results indicated that the production of TGF-β1 in CM-EV was significantly higher than that in ASC-EV. Therefore, it is thought that CM-EV exerted a greater influence on M2 polarization, resulting in a more effective therapeutic effect.

Latini et al. reported that miR-142 was significantly downregulated in SLE patients, while miR-155 also showed a trend of decreased expression [36]. MDM2, a protein involved in cell cycle regulation and immune modulation, is a common target of both miR-142 and miR-155 [36]. MDM2 was observed to be overexpressed in SLE patients, contributing to disease progression [36]. Their findings suggested that miR-142 and miR-155 play a crucial role in SLE by influencing MDM2 expression. Moreover, the therapeutic effect of mycophenolic acid was shown to be mediated by the upregulation of miR-142-3p/5p and miR-146a through histone modification at the promoter region, revealing a potential mechanism of action [37]. CM-EV showed a significantly higher expression of miR-142 and miR-155 than ASC-EV in this study. Therefore, it is believed that CM-EV exhibited better therapeutic effects (as observed in lupus-specific miRNA expression and survival rates) through the regulation of MDM2 and histone modification.

## Conclusions

CM-EV increased TGF-β1 production and miR-155-5p and miR-142-3p expression, resulting in increased survival, decreased anti-dsDNA antibody levels, and improved renal histopathology in a mouse model of SLE. Overall, these results suggest that EV produced from MSCs primed with conditioned media obtained from disease-conditioned immune cells, such as peripheral blood mononuclear cells of patients with SLE, exert immunomodulatory effects in the disease microenvironment.

## Supplementary Information

Supplementary Material 1.

## Data Availability

The datasets used and/or analyzed during the current study are available from the corresponding author on reasonable request.

## Abbreviations

ASC-EV: EV derived from the supernatant of iMSCs
BCA: Bicinchoninic acid
BUN: Blood urea nitrogen
C3: Complement component 3
CM-EV: EV derived from the supernatant of iMSCs primed with conditioned media obtained from disease-conditioned immune cells
CM-MSC: IMSCs primed with conditioned media obtained from disease-conditioned immune cells
Foxo: Forkhead box protein
IL: Interleukin
miR: MicroRNA
iMSCs: Immortalized MSCs
M1: CD11c+CD206- macrophages
M2: CD11c-CD206+ macrophages
MITF: Microphthalmia-associated transcription factor
MSC: Mesenchymal stem cells
NF-kB: Nuclear factor kappa light chain enhancer of activated B cells
NTA: Nanoparticle tracking analysis
NZB/W F1: New Zealand Black (NZB) x New Zealand White (NZW) F1
PI3K: Phosphatidylinositol-3 kinase
qRT-PCR: Quantitative real-time polymerase chain reaction
SLE: Systemic lupus erythematosus
SLEDAI: Systemic lupus erythematosus disease activity index
Th: T helper
Treg: Regulatory T cells
TUNEL: Terminal deoxynucleotidyl transferase dUTP nick-end labeling
