# Supplementary material for: Therapeutic effects of extracellular vesicles derived from mesenchymal stem cells primed with disease-conditioned-immune cells in systemic lupus erythematosus
Source: Arthritis Res Ther. 2024 Nov 18;26:201. doi: 10.1186/s13075-024-03435-1 (PMC11571884; doi:10.1186/s13075-024-03435-1)
Supplement: Supplementary file 1 — Supplementary Material 1. [file 13075_2024_3435_MOESM1_ESM.pdf]

Supplementary information

Therapeutic effects of extracellular vesicles derived from mesenchymal stem cells primed with disease-conditioned-immune cells in systemic lupus erythematosus

Eun Wha Choi[1,*], DVM, PhD, Il Seob Shin[1], DVM, PhD, I-Rang Lim[1], PhD, Jihye Lee[2], BS, Bongkum Choi[2], PhD, Sungjoo Kim[3,§], MD, PhD

[1]Department of Veterinary Clinical Pathology, College of Veterinary Medicine & Institute of Veterinary Science, Kangwon National University, 1 Kangwondaehak-gil, Chuncheon, Gangwon-do, 24341, Republic of Korea

[2]Bioanalysis Center, GenNBio Inc., 700, Daewangpangyo-ro, Bundang-gu, Seongnam-si, Gyeonggi-do, 13488, Republic of Korea

[3]GenNBio Inc., 80, Deurimsandan 2-ro, Cheongbuk-eup, Pyeongtaek-si, Gyeonggi-do, 17796, Republic of Korea

[§]Current address: Department of Surgery, Cheju Halla General Hospital, 65, Doryeong-ro, Jeju-si, Jeju-do, 63127, Republic of Korea

***Corresponding author:** Eun Wha Choi, DVM, PhD, Associate professor

Department of Veterinary Clinical Pathology, College of Veterinary Medicine & Institute of Veterinary Science, Kangwon National University, 1 Kangwondaehak-gil, Chuncheon, Gangwon-do, 24341, Republic of Korea

Telephone: 82-33-250-8794

Fax: 82-33-259-5625

E-mail: ewchoi@kangwon.ac.kr

Flow cytometry

Single cell suspensions were obtained from the spleens of NZB/W F1 mice at autopsy (at 43 weeks of age). An Fc blocking antibody was used to prevent non-specific binding (anti-mouse CD16/32, BioLegend, San Diego, CA, USA). To analyze T cell subset, the splenocytes were stained with peridinin chlorophyll protein complex-conjugated anti-mouse CD45 (PerCP-CD45, 1.25 μl/well, BioLegend), allophycocyanin-conjugated anti-mouse CD3e (APC-CD3e, 1 μl/well, eBioscience, San Diego, CA, USA), fluorescein isothiocyanate (FITC)-conjugated anti-mouse CD4 (FITC-CD4, 2 μl/well, BD Biosciences, San Jose, CA, USA), and PE-cyanine7-conjugated anti-mouse CD8a (0.5 μl/well, eBioscience).

Macrophage subset was analyzed; briefly, we examined proportion of M1 (CD45+ CD64+ CD11c+ CD206-) and M2 (CD45+ CD64+ CD11c- CD206+) cells using PerCP-conjugated anti-mouse CD45 (1.25 μl/well, BioLegend), APC-conjugated anti-mouse CD64 (5 μl/well, BioLegend), PE-conjugated anti-mouse CD11c (1.25 μl/well, BioLegend), and Alexa Fluor 488-conjugated anti-mouse CD206 (2 μl/well, BioLegend).

To analyze T helper cell subset, splenocytes were stained with antibodies to CD4 and CD25 (FITC-conjugated anti-mouse CD4 and APC-conjugated anti-mouse CD25, BD Biosciences). Cells were fixed and permeabilized prior to staining with PE-conjugated Foxp3 (0.5 μl/well, BD Biosciences), PE-conjugated ROR-γt (2 μl/well, ebioscience), PE-conjugated T-bet (2 μl/well, ebioscience), and PE-conjugated GATA-3 antibodies (4 μl/well, ebioscience).

Flow cytometry analysis was performed using a FACSymphony A3 (Becton Dickinson, Franklin Lakes, NJ, USA), and the data were analyzed using BD FACSDiva Software (BD).

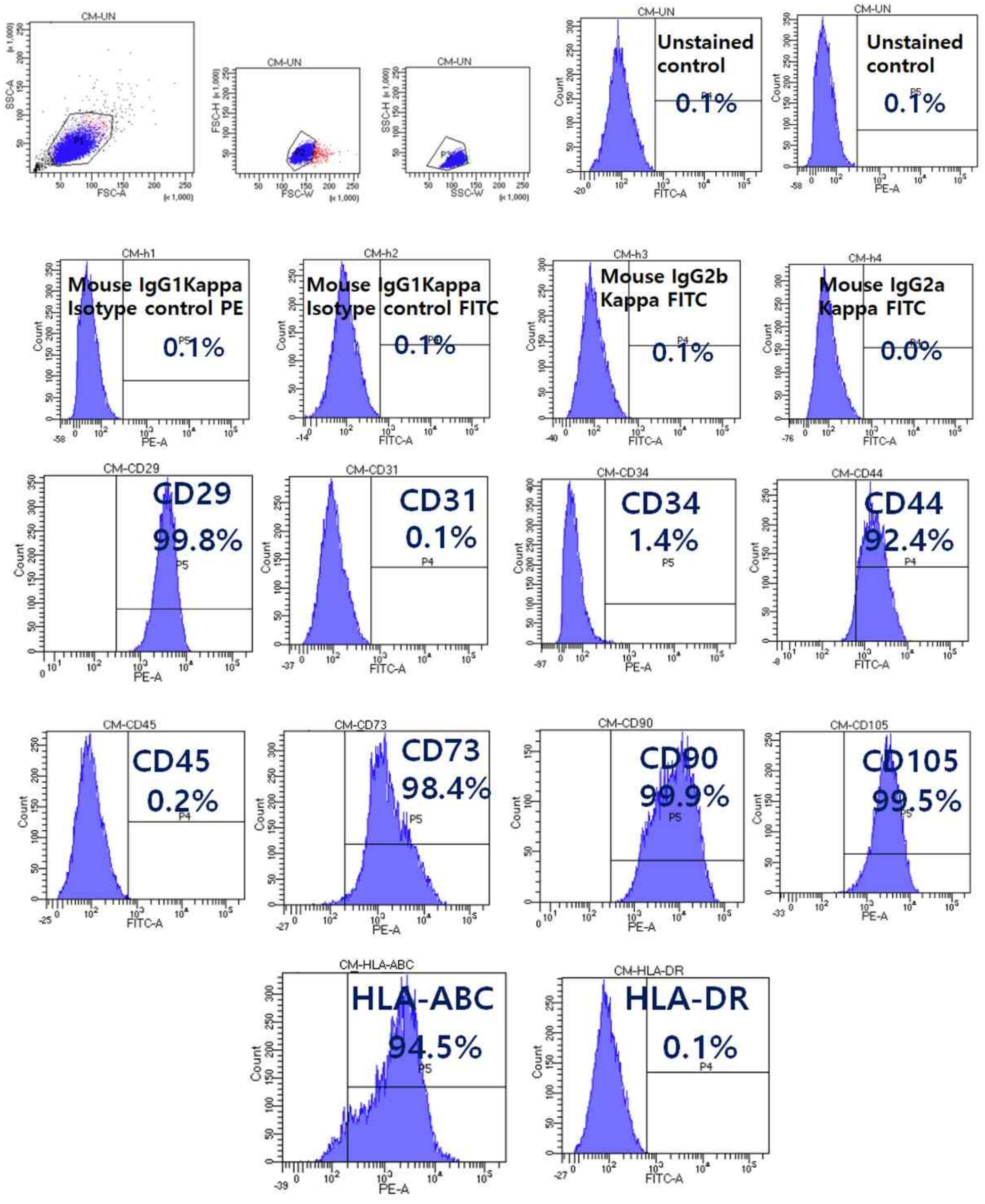

Supplementary Figure 1. Immunophenotype and differentiation capacity of immortalized mesenchymal stem cells primed with conditioned media obtained from disease-conditioned immune cells.

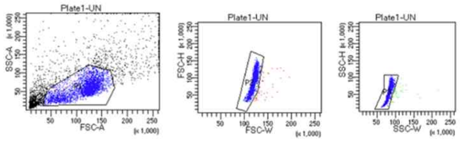

Unstained control

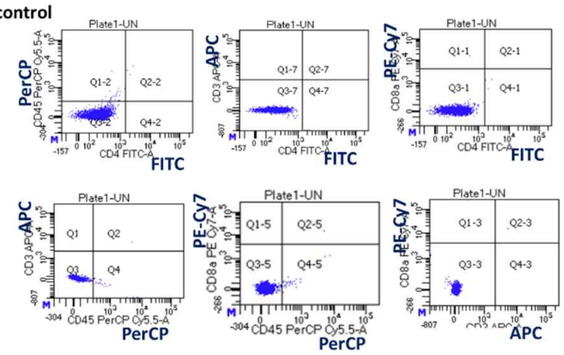

FITC-CD4 control

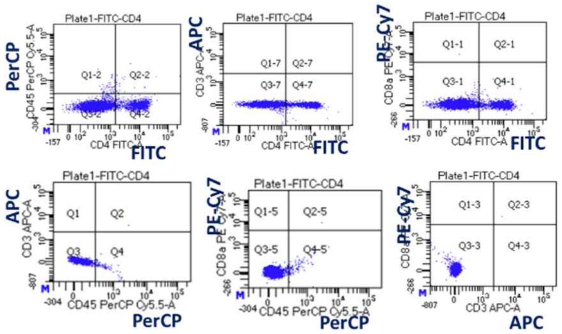

PE-Cy7-CD8a control

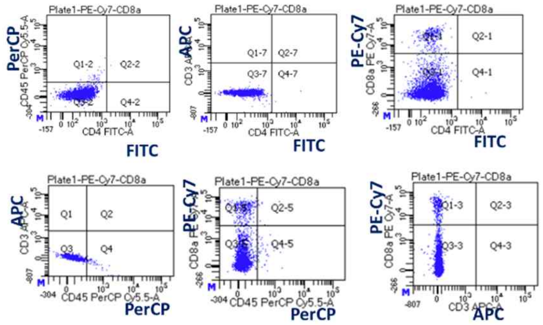

PerCP-CD45 control

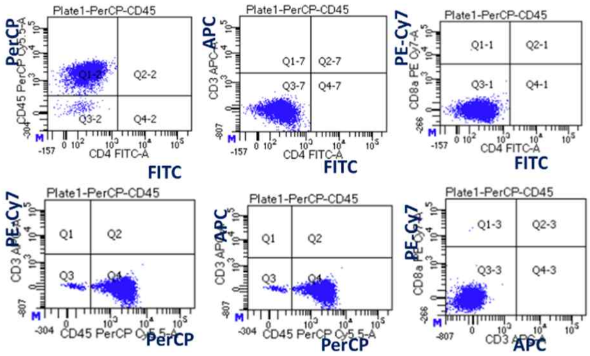

APC-CD3 control

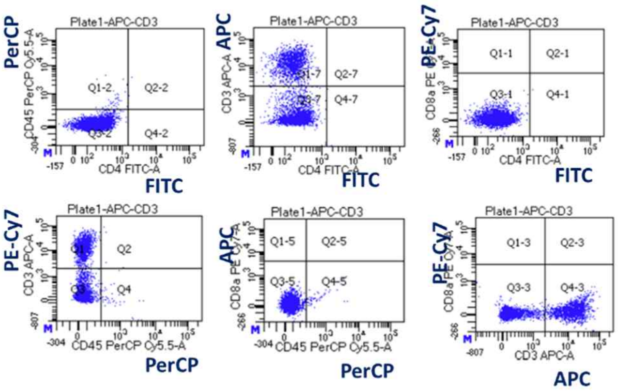

Isotype control

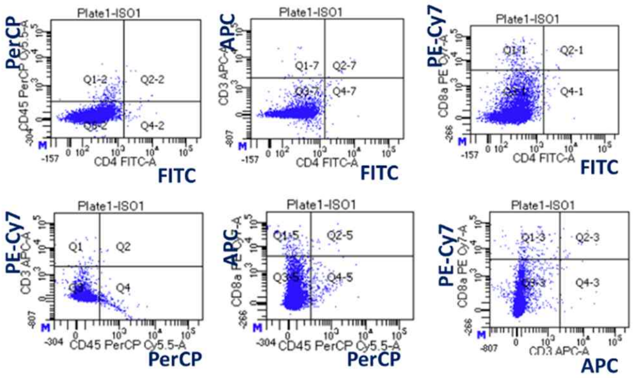

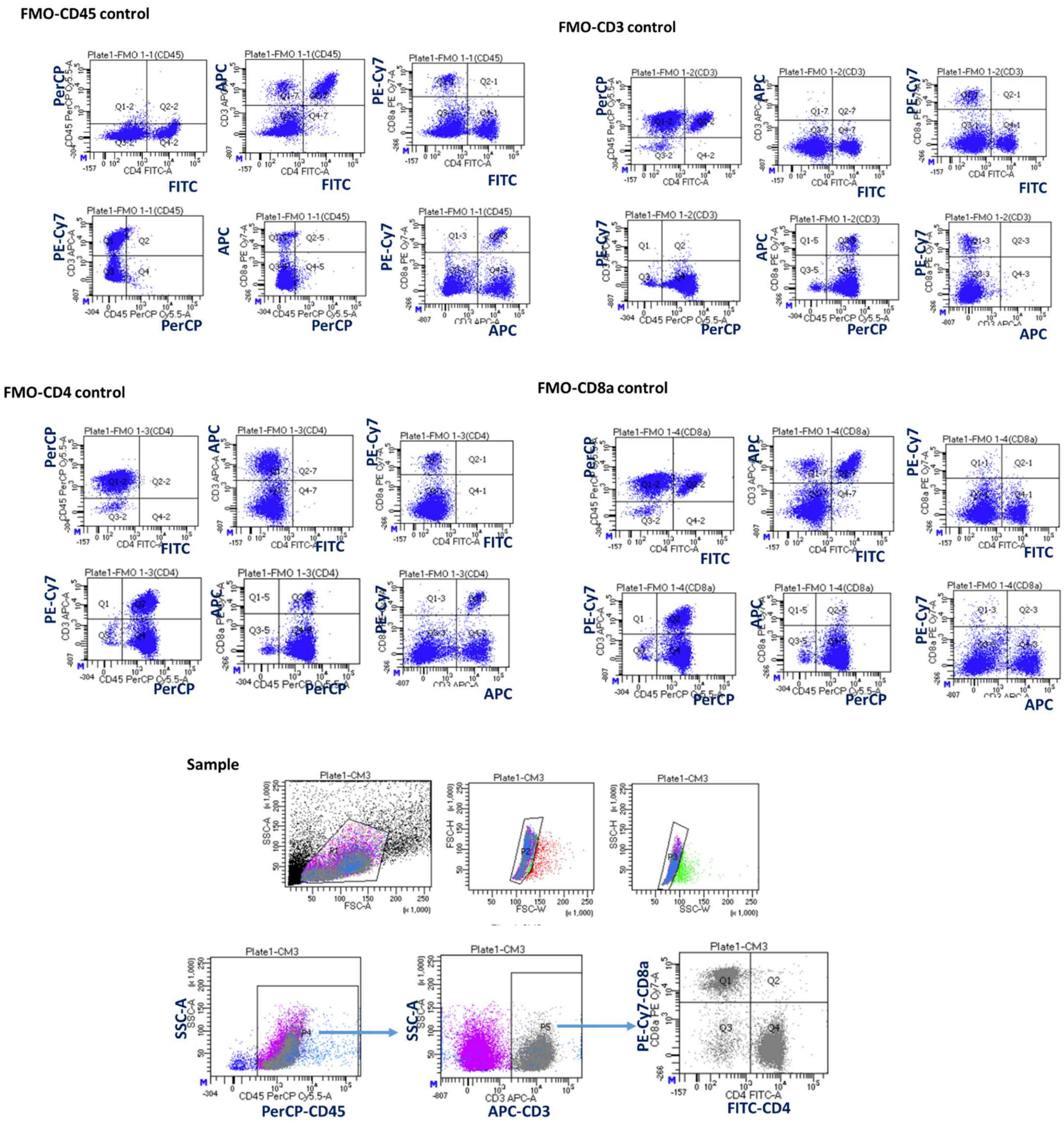

Supplementary Figure 2. A representative gating scheme of the T cell subset in the spleen.

Unstained control

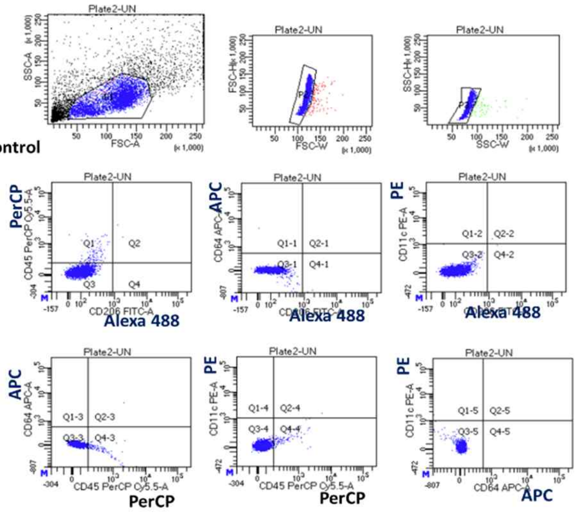

Alexa 488-CD206 control

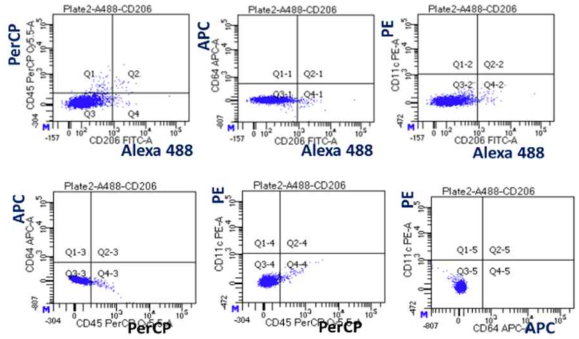

PerCP-CD45 control

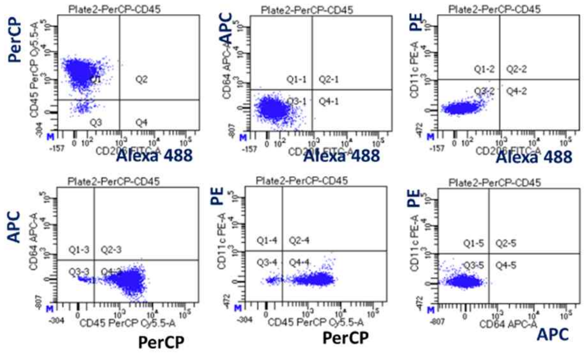

APC-CD64 control

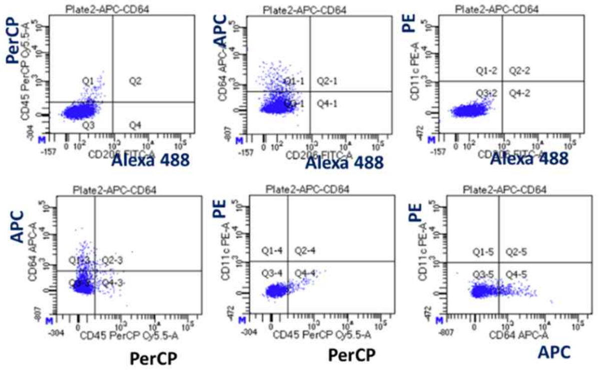

PE-CD11c control

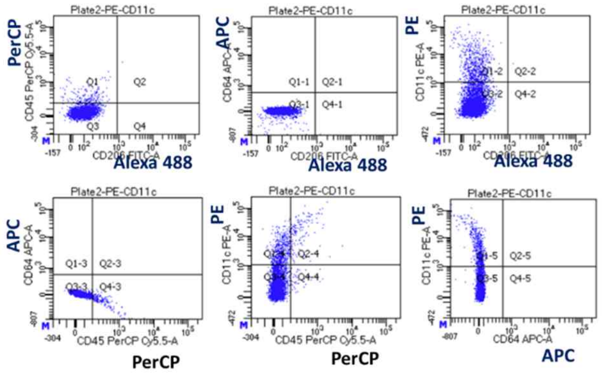

Isotype control

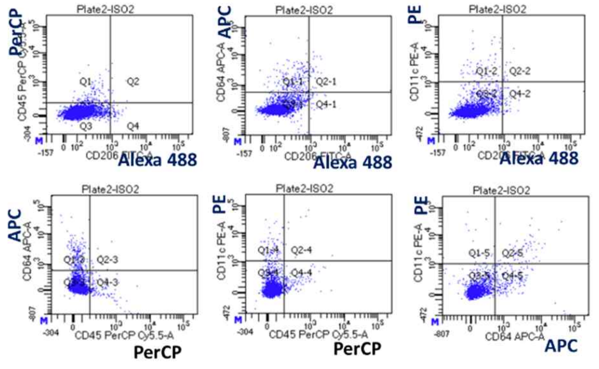

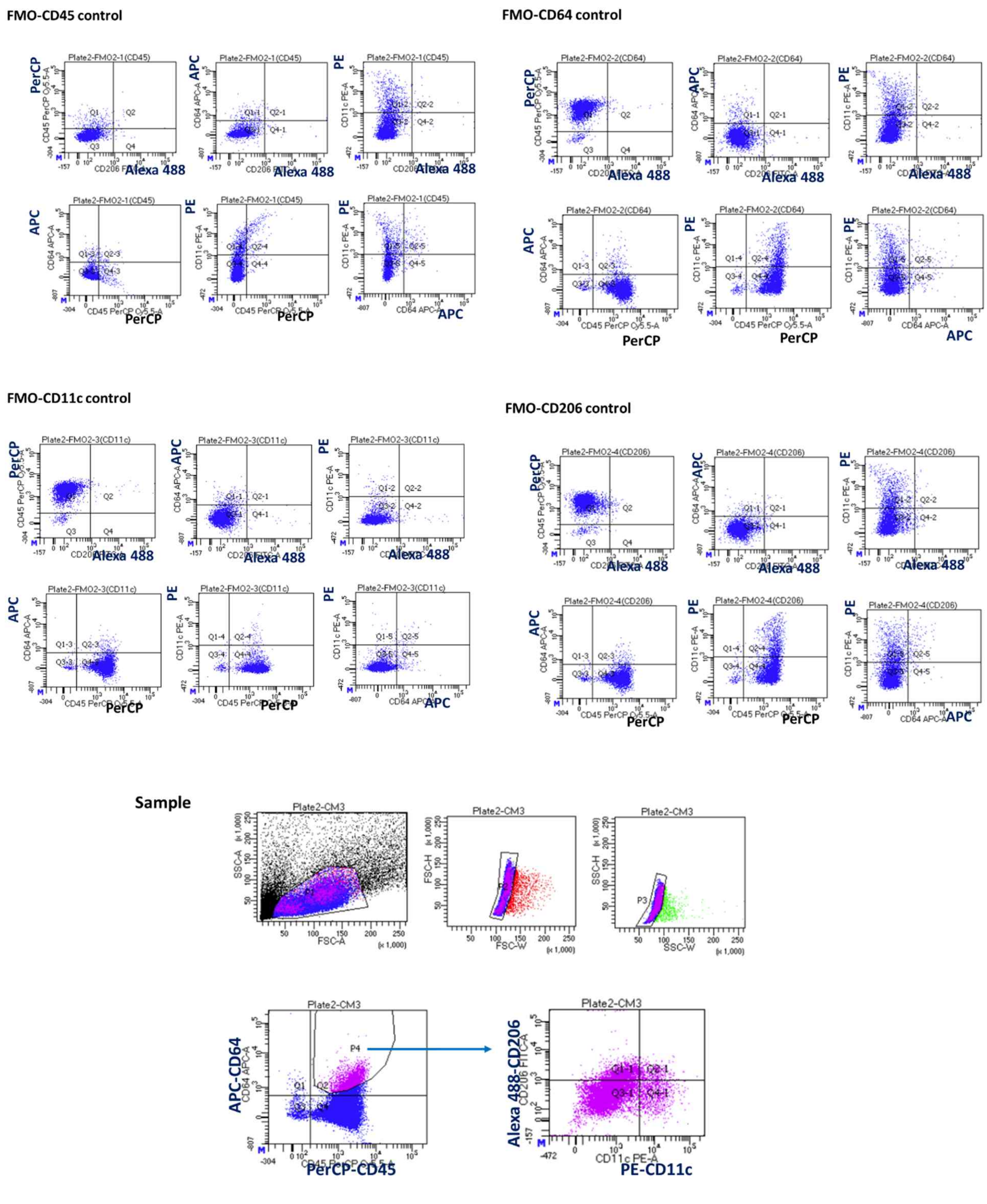

Supplementary Figure 3. A representative gating scheme of the macrophage subset in the spleen.

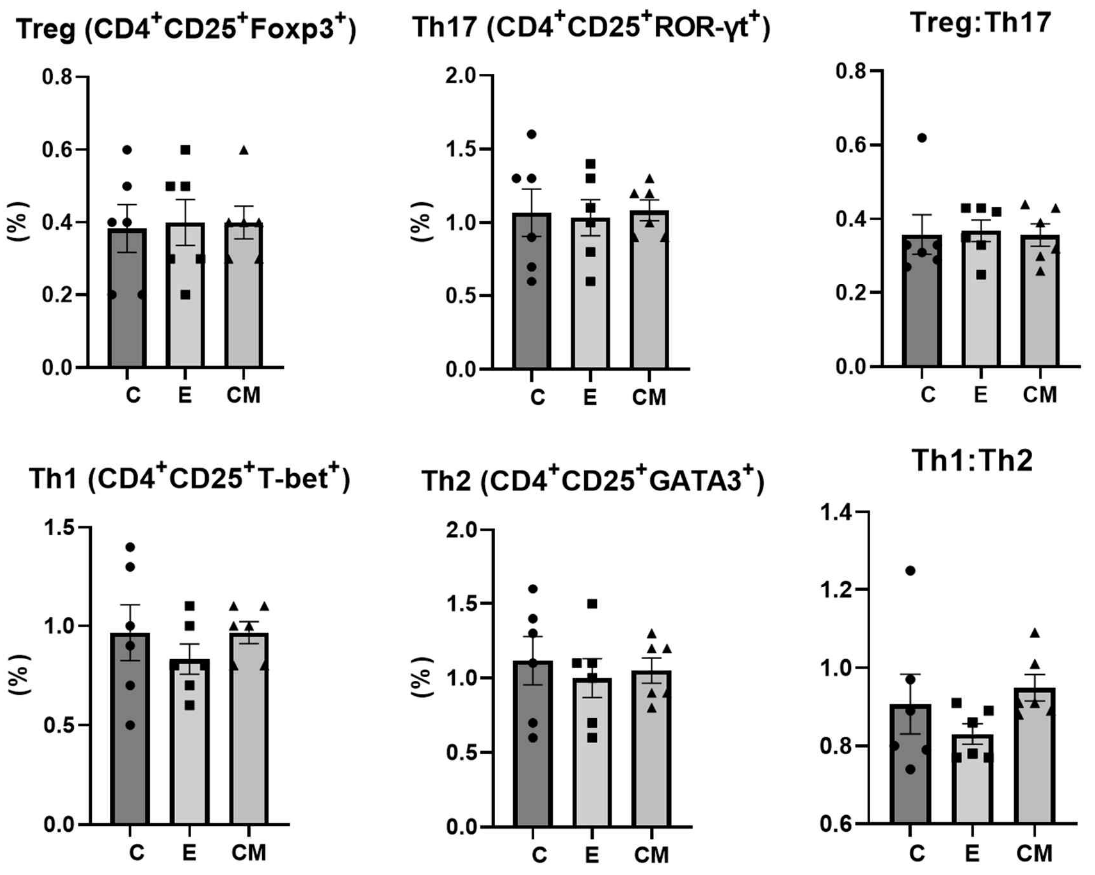

Supplementary Figure 4. The proportions of T helper cell subset in the spleen determined by flow cytometry. The proportions of $CD4^+CD25^+Fox3^+$ (Treg), $CD4^+CD25^+Fox3^+$ (Th17), Treg: Th17, $CD4^+CD25^+T\text{-}bet^+$ (Th1), and $CD4^+CD25^+GATA3^+$ (Th2) in the spleen (n = 6 per group). Data (mean ± standard error of mean) were compared using a one-way analysis of variance (†: $p < 0.05$) followed by Tukey's post-hoc tests. *: Significantly different compared with the control group (vs. group C, $p < 0.05$). No difference were observed. C: control (dPBS treatment group), E: ASC-EV treatment group and CM: CM-EV treatment group, Th: T helper, Treg: regulatory T cells.
